# Sex Hormone Receptor Signaling in Bladder Cancer: A Potential Target for Enhancing the Efficacy of Conventional Non-Surgical Therapy

**DOI:** 10.3390/cells10051169

**Published:** 2021-05-11

**Authors:** Hiroki Ide, Hiroshi Miyamoto

**Affiliations:** 1 Department of Urology, Keio University School of Medicine, Tokyo 160-8582, Japan; h-ide@fc4.so-net.ne.jp; 2 Department of Pathology & Laboratory Medicine, University of Rochester Medical Center, Rochester, NY 14642, USA; 3 Department of Urology, University of Rochester Medical Center, Rochester, NY 14642, USA; 4 James P. Wilmot Cancer Institute, University of Rochester Medical Center, Rochester, NY 14642, USA

**Keywords:** androgen receptor, estrogen receptor, bladder cancer, chemotherapy, BCG immunotherapy, radiotherapy, urothelial cancer

## Abstract

There have been critical problems in the non-surgical treatment for bladder cancer, especially residence to intravesical pharmacotherapy, including BCG immunotherapy, cisplatin-based chemotherapy, and radiotherapy. Recent preclinical and clinical evidence has suggested a vital role of sex steroid hormone-mediated signaling in the progression of urothelial cancer. Moreover, activation of the androgen receptor and estrogen receptor pathways has been implicated in modulating sensitivity to conventional non-surgical therapy for bladder cancer. This may indicate the possibility of anti-androgenic and anti-estrogenic drugs, apart from their direct anti-tumor activity, to function as sensitizers of such conventional treatment. This article summarizes available data suggesting the involvement of sex hormone receptors, such as androgen receptor, estrogen receptor-α, and estrogen receptor-β, in the progression of urothelial cancer, focusing on their modulation for the efficacy of conventional therapy, and discusses their potential of overcoming therapeutic resistance.

## 1. Introduction

Urinary bladder cancer, predominantly urothelial carcinoma, has been one of the commonly diagnosed malignancies, especially in men [1]. In addition, the number of deaths from bladder cancer throughout the world has risen from approximately 165,000 in 2012 [2] to 200,000 in 2018 [1]. Interestingly, in contrast to its incidence, the mortality rate among female patients with bladder cancer is higher [1,2]. Intravesical therapy with bacillus Calmette-Guérin (BCG), attenuated bacterial strains derived from Mycobacterium bovis, or chemotherapeutic agents, such as mitomycin C and doxorubicin, has been widely used in the management of non-muscle-invasive (NMI) bladder cancer after transurethral surgery [3]. In those with localized muscle-invasive (MI) bladder cancer, systemic cisplatin-based chemotherapy and/or radiotherapy have been employed before or after radical cystectomy [4,5,6,7]. Meanwhile, newly developed immunotherapy with immune checkpoint blockade has been expected to improve the survival of patients with advanced bladder cancer [5,6,7].

Sex hormone receptors, such as androgen receptor (AR), estrogen receptors (ERs) (i.e., ERα, ERβ), and progesterone receptor (PR), are a group of steroid receptors that are activated upon binding of cognitive ligands, androgens, estrogens, and progestogens, respectively. Recent findings indicate a vital role of sex hormone receptor signals in the pathogenesis of urothelial cancer, which may be the underlying reasons for the sex-related disparity in bladder cancer. Specifically, AR activation has been implicated in urothelial tumorigenesis, whereas conflicting results exist as to the estrogen effects that may be dependent on the functional activity of ERα versus ERβ in urothelial cells (reviewed in [8]). Particularly, it has been documented, using preclinical models for bladder cancer induced by known carcinogens in humans such as amine [9] and tobacco smoking [10], that AR knockdown results in strong prevention in its development. Moreover, emerging data suggest the association of androgen/estrogen-mediated receptor activity with not only the progression of urothelial tumors, but also the therapeutic efficacy in patients with bladder cancer. By contrast, to the best of our knowledge, no studies have demonstrated direct evidence to indicate the role of PR in bladder cancer [11], although an animal study [12] and a case–control study [13] have implied the preventive effects of progestogens on urothelial tumorigenesis. In this article, we mainly reviewed preclinical and clinical data implying the involvement of sex hormone receptors, particularly AR and ER signals, in modulating sensitivity to conventional non-surgical therapy for bladder cancer.

## 2. Sex Hormone Receptor Signaling and Bladder Cancer Progression

### 2.1. AR

AR expression in bladder cancer has been assessed by immunohistochemistry in surgical specimens. The overall immunoreactivity in bladder tumors has been reported to range from 13% to 54% [11]. Some of these studies have compared the rates of AR positivity in low-grade vs. high-grade tumors and/or NMI vs. MI tumors [14,15,16,17,18,19,20,21,22,23,24] (Table 1). Of them, four [14,17,18,24] and one [21] studies showed significant or insignificant down-regulation and significant up-regulation of AR expression, respectively, in high-grade tumors, while, in others [16,19,20,23], there were no significant differences in AR positivity between low-grade and high-grade tumors. Similarly, five [14,15,17,18,24] and two [16,21] studies showed significant or insignificant down-regulation and insignificant up-regulation, respectively, in MI tumors. Our meta-analysis published in 2017 [25] also demonstrated that AR positivity was significantly lower in high-grade tumors than in low-grade tumors [odds ratio (OR) = 0.575; 95% confidence interval (CI) = 0.421–0.785; *p* < 0.001], but that there was no significant difference between NMI and MI tumors (*p* = 0.356). These discordant findings on AR levels in various grades/stages of bladder cancer might be due to the use of different antibodies and/or protocols for staining as well as the lack of standardization in scoring. The prognostic value of AR expression in bladder tumors has additionally been assessed in some of these studies, showing conflicting findings. Specifically, AR positivity was significantly or insignificantly associated with better [17,20] and worse [18,23,24] outcomes, while two other studies [16,21] failed to show its prognostic significance. In our meta-analysis [25], AR expression in NMI bladder tumors was found to associate with better recurrence-free survival [hazard ratio (HR) = 0.593; 95% CI = 0.408–0.860; *p* = 0.006], but not with progression-free survival (*p* = 0.223).

The impact of androgens on tumor progression have been assessed in pre-clinical models for bladder cancer. Specifically, androgens, such as dihydrotestosterone (DHT) and methyltrienolone (R1881), induced cell proliferation, migration, and invasion in bladder cancer lines expressing a functional AR [9,26,27,28,29,30,31,32]. Correspondingly, gene silencing/knockdown of AR or treatment with AR antagonists, such as flutamide, bicalutamide, and enzalutamide, in these cell lines showed the inhibitory effects [9,26,27,28,29,30,31,33]. Epidermal growth factor (EGF) has also been shown to promote the growth of bladder cancer cells via the AR pathway [28]. Additionally, in mouse xenograft models for bladder cancer, AR inactivation resulted in the retardation of tumor growth [9,26,31]. In a transgenic mouse model where bladder cancer spontaneously developed, bilateral orchiectomy repressed tumor growth, which was restored by DHT supplementation [34].

**Figure 1:**
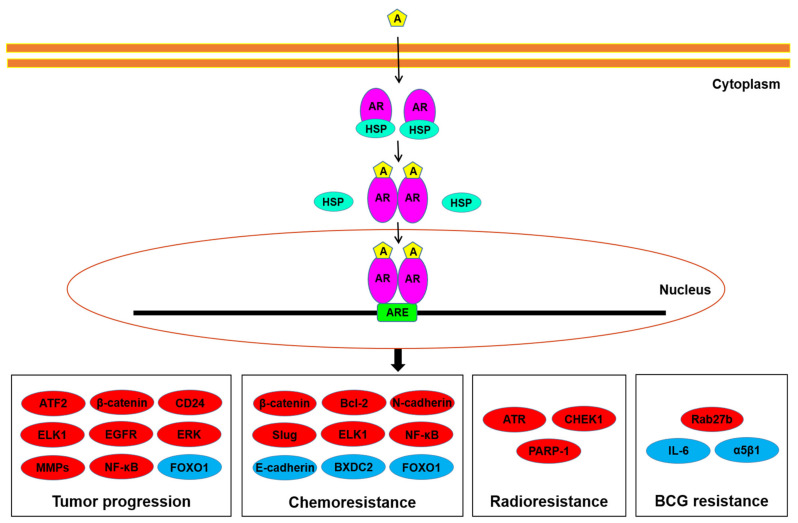
AR signaling in bladder cancer cells. Androgens have been suggested to induce tumor progression, as well as resistance to conventional non-surgical therapy, through the AR pathway via up-regulating (red) or down-regulating (blue) the molecules listed. A, androgen; ARE, androgen response element; HSP, heat shock protein.

Potential downstream targets of AR signals in bladder cancer cells have been explored (Figure 1). These molecules/pathways, some of which are transcription factors, include ATF2 [35], β-catenin/Wnt and its downstream c-myc and cyclin D1 [26,30,36], CD24 [29,33], EGF receptor (EGFR)/ERBB2/AKT/ERK [27], ELK1 [37], FOXO1 [32], IL-6 [31,38], matrix metalloproteinases (MMPs) [9,26,31,33], NF-κB [39], and vascular endothelial growth factor (VEGF) [9,33]. Indeed, most of these have been implicated in bladder cancer cell proliferation/apoptosis, cell invasion/metastasis, and/or epithelial-to-mesenchymal transition. These observations may thus represent underlying molecular mechanisms for how AR signals promote urothelial cancer progression.

### 2.2. ERs

As aforementioned, two classes of nuclear ERs exist in humans: ERα and ERβ encoded by *ESR1* and *ESR2*, respectively. The binding affinity of the major estrogen, 17β-estradiol (E2), for the ERα vs. ERβ is similar, while some ER agonists and antagonists preferentially bind to one (e.g., estrone/raloxifene to ERα) [40]. More importantly, these two receptors may have different biological functions.

The expression of ERα and ERβ in bladder tumors has been immunohistochemically investigated in surgical specimens. The overall positive rates of ERα and ERβ ranged from 1% to 38% and 22% to 100%, respectively [11,41]. Table 2 and Table 3 summarize the findings from studies comparing the rates of ERα/ERβ positivity in low-grade vs. high-grade tumors and/or NMI vs. MI tumors [17,18,20,41,42,43,44,45,46,47]. The expression of ERα was significantly down-regulated [18] or slightly up-regulated [41] in high-grade/MI tumors, while another study [42] showed no differences in its positivity. In addition, no prognostic role of ERα expression in bladder cancer has been documented [18,42]. Significant or insignificant up-regulation of ERβ expression in high-grade and/or MI tumors have been reported in more than half of the studies [18,20,43,44,47], whereas the other study [45] oppositely showed significant down-regulation. ERβ expression was also strongly associated with worse prognosis [17,18,20], while in one study [46], strong ERβ expression tended to be associated with better outcomes. In our meta-analysis [25], ERβ expression was found to be significantly up-regulated in high-grade (OR = 2.169; 95% CI = 1.583–2.971; *p* < 0.001) or MI (OR = 3.104; 95% CI = 2.081–4.631; *p* < 0.001) tumors, compared with low-grade or NMI tumors, respectively, and was associated with the risk of disease recurrence (HR = 1.573; 95% CI = 1.102–2.247; *p* = 0.013) or progression (HR = 2.236; 95% CI = 1.189–4.205; *p* = 0.089) in patients with NMI tumor.

Inconsistent data on the immunoreactivity for the ERα and ERβ in bladder tumor samples have thus been reported. Remarkably, the specificity of ER antibodies has been questioned [48,49]. In particular, only a few of commercially available anti-ERβ antibodies have been found to specifically target ERβ in immunostaining (and/or immunoblotting). Accordingly, many of the studies described above, including one [46] showing 100% positivity in a total of 313 tumors using PPG5/10, which has been found to not even target ERβ [48,49], as well as some of others, particularly with ERβ knockdown in cell lines described below, may not be credible.

The effects of ER ligands on tumor growth have been assessed, using preclinical models for bladder cancer. In ERα-positive bladder cancer lines, E2 induced cell proliferation, while selective ER modulators, including tamoxifen and raloxifene, as well as a pure ER antagonist ICI 182,780, inhibited it [45,50,51]. Tamoxifen and raloxifene have also been shown to inhibit the growth of ERα-negative/ERβ-positive bladder cancer cells and their xenograft tumors [44,51,52,53], while raloxifene failed to significantly affect that of ERα/ERβ knockdown lines [51]. Thus, activation of ER signals overall appears to be associated with the promotion of urothelial cancer progression. More specifically, selective agonists for ERα (i.e., propyl-pyrazole-triol) and ERβ (i.e., diatylpropionitrile) increased the cell proliferation of ERα-positive/ERβ-positive bladder cancer lines, but not those expressing an ERα-siRNA and an ERβ-siRNA, respectively [50]. Moreover, treatment with a selective ERβ antagonist (i.e., PHTPP) or knockdown of ERβ resulted in the suppression of cell growth [54,55]. By contrast, ERα knockdown was found to induce the growth of bladder cancer cells and their xenografts in mice [56], suggesting the inhibitory role of ERα in urothelial cancer progression. In accordance with these findings, a positive correlation between the expression of UGT1A, which was known to function as a tumor suppressor, and ERα levels in a urothelial cell line and bladder cancer specimens were observed, while UGT1A and ERβ expression was inversely correlated [57]. In addition, diatylpropionitrile could inhibit the migration and invasion of bladder cancer cells [58], suggesting the opposite function of ERβ in urothelial cancer. In addition, at least two prospective clinical trials have been conducted to assess the efficacy of tamoxifen in bladder cancer patients without (NCT02197897) or with (NCT00710970) prior chemotherapy, but no favorable effects have been reported.

**Figure 2:**
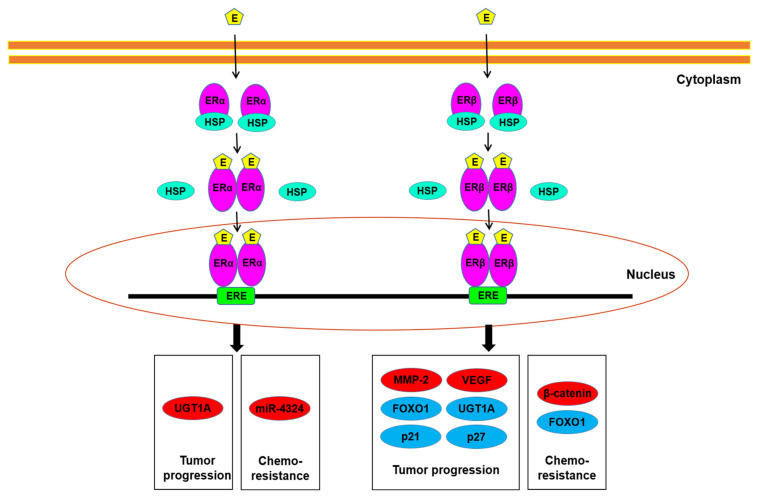
ER signaling in bladder cancer cells. Estrogens have been suggested to modulate tumor progression, as well as chemoresistance, through the ERα and/or ERβ pathways via up-regulating (red) or down-regulating (blue) the molecules listed. E, estrogen; ERE, estrogen response element; HSP, heat shock protein.

Underlying molecular mechanisms for ER function in bladder cancer have further been investigated (Figure 2). The oncogenic molecules/pathways potentially modulated by ER signaling in bladder cancer cells include AKT/ERK [50,55,56] and E-cadherin/N-cadherin [58], as well as MCM2 [59], which involves the initiation of DNA replication. The increase in apoptosis by raloxifene was also shown to mediate via inducing the cleavage of caspase-3 and BAD [51,52]. In our recent study described above [32], we demonstrated that ERβ could bind to the promoter region of FOXO1, a transcription factor functioning as a tumor suppressor, in bladder cancer cells, and that E2 treatment inactivated FOXO1 in ERα-negative/ERβ-positive cells, resulting in the up-regulation of MMP-2 and VEGF as well as down-regulation of p21 and p27. Recent studies have also indicated the link between ERα/ERβ activation and the modulation of microRNA, circular RNA, and enhancer RNA [60,61,62,63,64], all of which are known to involve bladder cancer progression.

## 3. Sex Hormone Receptor Signaling and Sensitivity to Conventional Non-Surgical Treatment for Bladder Cancer

Conventional non-surgical therapy against bladder cancer includes systemic chemotherapy, intravesical pharmacotherapy with anti-cancer agents or BCG, radiotherapy, and immunotherapy with immune checkpoint inhibitors. Although these are quite effective in some patients, bladder cancer, especially MI disease, remains lethal [65]. The development of strategies for not only overcoming therapeutic resistance where underlying mechanisms are poorly understood, but also predicting its sensitivity, is thus urgently required. Meanwhile, sex hormone receptor signals have been implicated in modulating sensitivity to conventional therapy for bladder cancer. The main findings in preclinical studies [22,30,32,37,55,66,67,68,69,70,71,72,73,74,75,76,77,78,79] suggesting this are summarized in Table 4.

### 3.1. Chemotherapy

Cisplatin-based combination chemotherapy (e.g., MVAC: methotrexate/vinblastine/doxorubicin/cisplatin; GC: gemcitabine/cisplatin) remains the standard of care in patients with locally advanced or metastatic bladder cancer. These regimens are also widely used prior to radical cystectomy as neoadjuvant therapy. However, a considerable number of patients either fail to respond or eventually acquire resistance. In addition, several chemotherapeutic agents, such as doxorubicin, mitomycin C, and thiotepa, have been given intravesically to those with superficial bladder tumor following transurethral surgery primarily as prophylactic therapy.

In bladder cancer specimens from patients who had subsequently received cisplatin-based neoadjuvant chemotherapy, there was a correlation between AR immunoreactivity and chemosensitivity (i.e., AR-positive in 21% of responders vs. 45% of non-responders, *p* = 0.087) [72]. In bladder cancer sublines resistant to cisplatin [71,80], as well as gemcitabine [72], AR expression has been found to be considerably elevated, compared to control or parental cells. These findings suggest the involvement of AR signals in chemoresistance in bladder cancer.

Cell proliferation assay data indeed showed that bladder cancer lines with AR overexpression [75] or androgen treatment [71] were more resistant to cisplatin. Correspondingly, AR silencing/knockdown or antagonist (e.g., hydroxyflutamide, enzalutamide) treatment enhanced the cytotoxic effects of cisplatin in bladder cancer cells, even resistant sublines [22,71]. Enzalutamide was also shown to induce apoptosis and prevent cell migration/invasion in the presence of cisplatin [22]. Mechanistically, enzalutamide treatment was associated with increases in the expression of BAX, cleaved caspase-3, cleaved PARP, and an epithelial marker E-cadherin, and decreases in that of Bcl-2 and mesenchymal markers (e.g., β-catenin, N-cadherin, Slug, vimentin), although there appeared to be no significant differences in their expression between cisplatin + enzalutamide vs. cisplatin or enzalutamide alone [22].

Similar findings have been reported with other anti-cancer agents. Specifically, AR-positive bladder cancer cells with DHT treatment or AR silencing were shown to be less or more, respectively, sensitive to doxorubicin, compared with controls [30]. Additionally, in a gemcitabine-resistant bladder cancer subline, enzalutamide was found to restore its sensitivity while reducing the expression of cyclin D1 [72]. A more recent study demonstrated that ASC-J9^®^, an AR degradation enhancer, could increase sensitivity to not only cisplatin and doxorubicin, but also mitomycin C in AR-positive bladder cancer cells [55]. These findings indicate that activation of AR signaling is associated with chemoresistance in bladder cancer.

We have further explored how AR signals modulate chemosensitivity. There were close correlations of AR expression/activity with those of NF-κB, which is considered to be a key molecule for cisplatin resistance, in bladder cancer cells [39,72]. We have also found that androgen up-regulates the expression of a *c*-*fos* proto-oncogene regulator ELK1 in bladder cancer cells [37] and that ELK1 inactivation via stable expression of a shRNA or treatment with a selective α1-blocker silodosin increases sensitivity to cisplatin [68]. Immunohistochemistry in surgical specimens form patients subsequently undergoing cisplatin-based neoadjuvant chemotherapy, phospho-ELK1 positivity was significantly (*p* = 0.039) higher in those from non-responders (71%) than in those from responders (38%) [68]. We recently demonstrated that androgen/AR could down-regulate the expression of BXDC2, also named BRIX1, which involves ribosome biogenesis, in bladder cancer cells, and loss of BXDC2 in cell lines and surgical specimens was associated with cisplatin resistance [79]. Furthermore, an ERK activator reduced BXDC2 expression in bladder cancer cells, while BXDC2 knockdown failed to affect phospho-ERK expression [79], suggesting cisplatin resistance via the AR → ERK → BXDC2 signaling pathway.

Similar to our immunohistochemistry data on AR [71], we recently demonstrated that the rate of ERβ positivity in transurethral resection specimens was significantly lower in responders to cisplatin-based neoadjuvant chemotherapy than in non-responders (37% vs. 71%, *p* = 0.016), especially in female patients (20% of responders vs. 100% of non-responders, *p* = 0.048), but not in males (42% of responders vs. 65% of non-responders, *p* = 0.142) [76]. Meanwhile, elevated ERβ expression in adjacent normal bladder tissues was strongly associated with a worse prognosis in patients undergoing cisplatin-based chemotherapy [74]. Two early studies in bladder cancer lines by one group showed that treatment with tamoxifen, along with methotrexate, vinblastine, doxorubicin, cisplatin, mitomycin C, or thiotepa, more strongly inhibited their proliferation, compared to that with each chemotherapeutic drug alone [66,67]. However, in these assays, the combination effects were not directly compared with that of tamoxifen alone, and it might therefore be unable to conclude that tamoxifen could increase sensitivity to each cytotoxic agent. The same group conducted a clinical trial of MVAC plus a high dose (200 mg/m^2^/day, days 1–4) of tamoxifen in 30 patients with advanced bladder cancer, showing the comparable response rate to that known for conventional MVAC therapy, but no control arm with MVAC alone was compared [81]. In an additional in vitro study, gemcitabine combined with tamoxifen showed stronger inhibitory effects on the growth of bladder cancer cells than gemcitabine or tamoxifen alone [70], but the rates of inhibition by tamoxifen in the absence versus presence of gemcitabine were not directly compared. In a recent study, co-culture of cancer-associated fibroblasts was shown to induce ERβ expression in bladder cancer cells while reducing the cytotoxicity of cisplatin [74]. We further demonstrated that tamoxifen treatment or ERβ knockdown in ERα-negative bladder cancer cells resulted in the enhancement of cisplatin sensitivity [76]. Moreover, in the cisplatin-resistant sublines, ERβ expression was considerably elevated, while E2 induced the expression and activity of β-catenin which was known to involve cisplatin resistance [76]. Thus, activation of ER, especially ERβ, is likely associated with chemoresistance. Additionally, in a study showing that ERα could induce the expression of miR-4324 via binding to its promoter in bladder cancer cells, overexpression of miR-4324 significantly induced the cytotoxic effects of doxorubicin [63], suggesting an association between ERα activation and increased sensitivity to doxorubicin.

As aforementioned, both androgen and estrogen could inactivate a tumor suppressor FOXO1 via the AR and ERβ pathways, respectively, in bladder cancer cells [32]. We further found that silencing of FOXO1 or treatment with an FOXO1 inhibitor in bladder cancer cells resulted in the reduction of sensitivity to cisplatin [77]. The expression of an inactivated form phospho-FOXO1 was considerably up-regulated in cisplatin-resistant cells, compared with control cells, and phospho-FOXO1 expression in transurethral resection specimens from patients undergoing cisplatin-based neoadjuvant chemotherapy was more often seen in non-responders (67.9%) than in responders (38.9%) [77]. FOXO1 inactivation could thus be an underlying mechanism for chemoresistance in bladder cancer induced by AR and/or ERβ signals.

A phase 1/1b clinical trial has been conducted to assess if AR modulation enhances the efficacy of chemotherapy (NCT02300610; completed in December 2012). In a total of 10 patients with urothelial cancer receiving standard doses of gemcitabine and cisplatin, oral enzalutamide (80 or 160 mg) was added. Although some of the patients with 160 mg enzalutamide showed partial response, no control arm with no enzalutamide treatment was compared.

### 3.2. Radiotherapy

In selected patients with MI bladder cancer, radiotherapy combined with chemotherapy led to survival rates comparable to those undergoing radical cystectomy [4]. Specifically, in these patients, trimodal therapy consisting of either transurethral resection or partial cystectomy followed by radiotherapy with concurrent chemotherapy is currently considered to yield the best oncologic outcomes [4,65]. Although the trimodal therapy may increase quality-adjusted life years, current data on overall survival or disease-specific survival are still in favor of radical cystectomy [82]. Thus, the development of the novel radiosensitization strategies is required to replace radical cystectomy with radiotherapy as a gold standard option for some MI bladder cancers.

We showed that bladder cancer lines endogenously or exogenously expressing a full-length wild-type human AR were significantly less sensitive to irradiation, compared with AR knockdown or control AR-negative sublines, respectively [73]. Correspondingly, DHT or hydroxyflutamide treatment in AR-positive bladder cancer lines significantly reduced or induced, respectively, the cytotoxic effects of irradiation. Meanwhile, radiation-resistant sublines established following 2 Gy ionizing radiation six times/two weeks showed significant elevation in the expression of not only DNA repair genes, such as *ATR*, *CHEK1*, and *PARP-1*, but also AR mRNA/protein. In mouse xenograft models for bladder cancer, considerable increases in radiosensitivity by AR knockdown or anti-androgen treatment were verified. Mechanistically, AR inactivation via knockdown or hydroxyflutamide treatment was found to be associated with a delay in DNA double-strand break repair (e.g., γH2AX resolution) 4–24 h after irradiation. Additionally, in irradiated AR-positive cells, DHT induced the expression of the DNA repair genes, which was restored by hydroxyflutamide. Our findings suggest that AR activity is inversely associated with radiosensitivity in bladder cancer and that concurrent androgen deprivation may function as a sensitizer of irradiation, especially in patients with AR-positive tumor.

In a recent prospective trial (NCT04282876; started in February 2020), patients with MI bladder cancer undergoing radiotherapy are being recruited. A group of these patients is randomized to simultaneously receive a gonadotropin-releasing hormone antagonist (i.e., degarelix) as chemical castration. Primary outcome measures include bladder fibrosis 3 months after irradiation, but oncologic outcomes will not appear to be compared.

### 3.3. Immunotherapy

Intravesical BCG immunotherapy has been widely used for the treatment of urothelial carcinoma in situ and the prevention of disease recurrence after transurethral resection of NMI tumors. Interestingly, although data are conflicting, male [83] or female [84] bladder cancer patients have been shown to be considerably less sensitive to BCG therapy, suggesting the involvement of sex hormone receptor signals in BCG sensitivity.

In an earlier study, DHT was found to inhibit the expression and transactivation of IL-6 induced by BCG treatment in bladder cancer cells [38]. Another study showed that hydroxyflutamide and ASC-J9 increased the expression level of BCG-mediated integrins (e.g., α5β1) and intake of BCG in bladder cancer cells, as well as recruitment of monocytes/macrophages [69]. BCG, along with each AR inhibitor, also more strongly inhibited the growth of bladder cancer cells and chemical carcinogen N-butyl-N-(4-hydroxybutyl)nitrosamine-induced bladder tumors in mice, compared with BCG or AR inhibitor alone [69]. These findings implied that AR signaling might contribute to modulating sensitivity to BCG therapy. We then demonstrated direct evidence to indicate the link between AR activation and BCG resistance [78]. AR knockdown or overexpression in bladder cancer lines was associated with considerable induction or reduction, respectively, in intracellular BCG quantity and BCG cytotoxicity. AR expression was considerably higher in BCG-resistant bladder cancer cells following repeating exposure to BCG for over six months, compared with control cells, and AR positivity immunohistochemically determined in NMI bladder cancer specimens from patients who had subsequently undergone intravesical BCG immunotherapy was strongly associated with worse outcomes, compared to those with AR-negative tumor. We also performed DNA microarray screening and identified Rab27b, a small GTPase known to mediate bacterial exocytosis, which was up-regulated in BCG-resistant cells and down-regulated in AR knockdown cells. Indeed, knockdown/overexpression of Rab27b or its known effector SYTL3, as well as treatment with GW4869 known to inhibit Rab27b-dependent secretion, was found to considerably modulate BCG quantity in bladder cancer cells, as well as its cytotoxicity in vitro and in vivo. In addition, Rab27b positivity in the same cohort of patients with BCG therapy was associated with a significantly higher risk of tumor recurrence. Thus, our findings suggest that AR signaling reduces the efficacy of BCG therapy, presumably via modulating Rab27b-induced exocytosis in bladder cancer cells.

The impact of ER signaling on the efficacy of BCG therapy in bladder cancer has also been investigated. In ERα-positive/ERβ-positive bladder cancer cells, E2 reduced BCG attachment and internalization as well as monocyte/macrophage recruitment, whereas tamoxifen and a pure anti-estrogen ICI 182,780 reversed the estrogen effect [85]. These anti-estrogens were also found to enhance the cytotoxic effects of BCG in cell line and mouse models for bladder cancer [85]. In addition, a phase 2 randomized clinical trial is ongoing to assess if genistein, a biologically active isoflavone and a phytoestrogen with structure similar to that of E2, not only helps alleviate the adverse of intravesical BCG therapy but also improves its efficacy (NCT01489813; started in May 2017). In this study, either genistein supplement or placebo is given to the patients with NMI bladder tumor for 10 weeks (i.e., during BCG therapy and one-month post-therapy). Several antibodies against programmed cell death-1 (PD-1) or its ligand (PD-L1) have recently been approved by the U.S. Food and Drug Administration, and there are a number of PD-1/PD-L1 inhibitors entering clinical trials [65]. These drugs, as immune checkpoint inhibitors that attack tumor cells via enhancing the host immune response, are expected to considerably improve the prognosis of various types of malignancies, including bladder cancer. Importantly, the efficacy of PD-1/PD-L1 inhibitors is often associated with the levels of PD-L1 expression [86]. In an immunohistochemical study in MI bladder cancers, PD-L1 expression was shown to be inversely correlated with the levels of AR expression [87]. We have confirmed this inverse correlation in bladder cancer cell lines (Teramoto and Miyamoto, unpublished data). In breast cancer specimens, an inverse correlation of ERα status with *PD-L1* mRNA expression has also been documented [88].

## 4. Conclusions

Current evidence indicates a critical role of sex hormone receptor signaling in bladder cancer progression, supporting that urothelial cancer is an endocrine-related neoplasm. However, it remains uncovered how AR and ERs function in urothelial cancer cells. Various studies have also suggested the involvement of sex hormone receptors in modulating sensitivity to conventional non-surgical therapy for bladder cancer. Specifically, activation of AR and ER signals appears to be associated with resistance to chemotherapy, radiotherapy, and BCG immunotherapy, although limited data, especially those on ERα, are available. Accordingly, concurrent inactivation of these, using, for example, anti-AR or anti-ER agents widely used for the treatment of other pathologic conditions such as prostate and breast cancers, is anticipated to improve patient outcomes via sensitizing the efficacy of the conventional therapy, in addition to direct inhibitory effects of androgen/estrogen deprivation. Meanwhile, eventual resistance to standard hormonal therapy remains a critical issue in patients with prostate or breast cancer. Indeed, an AR variant, which is implicated in the development of castration-resistant prostate cancer, has recently been identified in bladder cancer [89]. Further investigation of AR and ERs, as well as other molecules directly or indirectly regulated by AR/ER signals, is required for determining the precise actions of androgens/estrogens in bladder cancer cells, in relation to their impact on modulating sensitivity to conventional therapy, as well as underlying molecular mechanisms for their actions.

## Figures and Tables

**Table 1:** Immunoreactivity for AR in low-grade vs. high-grade and NMI vs. MI bladder cancers and its prognostic significance.

Author, Year [Ref]	Tumor Grade			Tumor Stage			Prognostic Significance
	Low-Grade	High-Grade	*p* ^a^	NMI	MI	*p* ^a^	
Boorjian, 2004 [14]	8/9 (89%)	16/33 (48%)	0.055	21/28 (75%)	3/14 (21%)	0.002	NA
Boorjian, 2009 [15]	NA	NA	NA	13/22 (59%)	11/33 (33%)	0.095	NA
Mir, 2011 [16]	11/90 (12%)	50/382 (13%)	0.864	11/126 (9%)	46/305 (15%)	0.086	NS
Tuygun, 2011 [17]	46/72 (64%)	25/67 (37%)	0.002	64/106 (60%)	7/33 (21%)	<0.001	*p* = 0.095 (RFS/NMI)
Miyamoto, 2012 [18]	31/56 (55%) ^b^	48/132 (36%)	0.023	49/97 (51%)	30/91 (33%)	0.018	*p* = 0.0705 (PFS/MI)
Jing, 2014 [19]	22/40 (55%)	9/18 (50%)	0.781	22/45 (49%)	9/13 (69%)	0.225	NA
Nam, 2014 [20]	47/120 (39%) ^b^	16/49 (33%)	0.485	NA	NA	NA	*p* = 0.001 (RFS)
Elzamy, 2018 [21]	7/48 (15%) ^b^	30/58 (52%)	<0.001	5/27 (19%)	32/79 (41%)	0.060	NS
Tyagi, 2019 [22]	NA	NA	NA	17/38 (45%)	22/34 (65%)	0.103	NA
Yonekura, 2019 [23]	11/26 (42%)	9/14 (64%)	0.320	NA	NA	NA	*p* < 0.05 (RFS&PFS/NMI)
Toren, 2020 [24]	76/121 (63%) ^c^	79/194 (41%) ^c^	<0.001	95/150 (63%)	61/167 (37%)	<0.001	*p* = 0.03 (RFS/RC)

NMI, non-muscle-invasive; MI, muscle-invasive; NA, not available; NS, not significant; RFS, recurrence-free survival; PFS, progression-free survival; RC, radical cystectomy cases. ^a^ We calculated two-tailed *p* values, using Fisher’s exact test. ^b^ Cases of papillary urothelial neoplasm of low malignant potential (PUNLMP) are included. ^c^ Low-grade = Grades 1–2 vs. High-grade = Grade 3.

**Table 2:** Immunoreactivity for ERα in low-grade vs. high-grade and NMI vs. MI bladder cancers and its prognostic significance.

Author, Year [Ref]	Tumor Grade			Tumor Stage			Prognostic Significance
	Low-Grade	High-Grade	*p* ^a^	NMI	MI	*p* ^a^	
Miyamoto, 2012 [18]	21/56 (38%) ^b^	30/132 (23%)	0.048	34/97 (35%)	17/91 (19%)	0.014	NS
Imai, 2019 [41]	20/63 (32%) ^c^	28/62 (45%) ^c^	0.143	26/81 (32%)	22/44 (50%)	0.056	NA
Bernardo, 2020 [42]	2/12 (17%)	12/68 (18%)	1.000	7/40 (18%)	7/40 (18%)	1.000	NS

NMI, non-muscle-invasive; MI, muscle-invasive; NS, not significant; NA, not available. ^a^ We calculated two-tailed *p* values, using Fisher’s exact test. ^b^ Cases of papillary urothelial neoplasm of low malignant potential (PUNLMP) are included. ^c^ Low-grade = Grades 1–2 vs. High-grade = Grades 3–4.

**Table 3:** Immunoreactivity for ERβ in low-grade vs. high-grade and NMI vs. MI bladder cancers and its prognostic significance.

Author, Year [Ref]	Tumor Grade			Tumor Stage			Prognostic Significance
	Low-Grade	High-Grade	*p* ^a^	NMI	MI	*p* ^a^	
Croft, 2005 [43]	6/50 (12%) ^b^	14/42 (33%) ^b^	0.021	NA	NA	NA	NA
Shen, 2006 [44]	66/114 (58%) ^b^	67/96 (70%) ^b^	0.085	78/145 (54%)	47/59 (80%)	<0.001	NA
Kontos, 2010 [45]	54/57 (95%) ^b^	30/54 (56%) ^b^	<0.001	25/30 (83%)	22/41 (54%)	0.011	NA
Tuygun, 2011 [17]	16/72 (22%)	21/67 (31%)	0.253	28/106 (26%)	12/33 (36%)	0.279	*p* = 0.035 (PFS/NMI)
Miyamoto, 2012 [18]	16/56 (29%) ^c^	77/132 (58%)	<0.001	39/97 (34%)	60/91 (66%)	<0.001	*p* < 0.01 (PFS/NMI); *p* < 0.01 (PFS&CSS/MI)
Nam, 2014 [20]	32/120 (27%) ^c^	20/49 (41%)	0.098	NA	NA	NA	*p* < 0.05 (RFS&PFS)
Tan, 2015 [46]	28/28 (100%) ^d^	262/262 (100%) ^d^	1.000	95/95 (100%)	216/216 (100%)	1.000	*p* = 0.055–0.087 (CSS)
Nguyen, 2017 [47]	2/6 (33%)	16/24 (67%)	0.184	3/11 (27%)	15/19 (79%)	0.009	NS
Bernardo, 2020 [42]	11/12 (92%)	62/68 (91%)	1.000	36/40 (90%)	37/40 (93%)	1.000	NA

NMI, non-muscle-invasive; MI, muscle-invasive; NA, not available; NS, not significant; RFS, recurrence-free survival; PFS, progression-free survival; CSS, cancer-specific survival. ^a^ We calculated two-tailed *p* values, using Fisher’s exact test. ^b^ Low-grade = Grades 1–2 vs. High-grade = Grade 3. ^c^ Cases of papillary urothelial neoplasm of low malignant potential (PUNLMP) are included. ^d^ Low-grade = Grades 1–2 vs. High-grade = Grades 3–4.

**Table 4:** Preclinical studies suggesting the involvement of AR/ER signaling in modulating sensitivity to conventional therapy for bladder cancer.

Author, Year [Ref]	Conventional Therapy	Receptor	Design/Model	Main Findings	Molecules/Pathways Involved
Pu, 1995 [66]	CT (CIS, DOX, MTX, VBL)	ER	T24/NTUB1/BFTCC905 cell viability	TAM ↑growth inhibition	NA
Pu, 1996 [67]	CT (DOX, MMC, TTP)	ER	TSGH8301/HTB9 cell viability	TAM ↑growth inhibition	NA
Shiota, 2012 [30]	CT (DOX)	AR	UMUC3 cell viability	DHT ↓sensitivity AR-siRNA ↑sensitivity	NA
Kawahara, 2015 [37] Kawahara, 2015 [68]	CT (CIS)	AR	UMUC3 cell viability	DHT ↑ELK1 ELK1-inactivation ↑sensitivity	ELK1
Shang, 2015 [69]	BCG	AR	253J/T24 cell viability BBN-induced tumor in mice	ASC-J9/HF ↑growth inhibition	integrin α5β1
Takeuchi, 2015 [70]	CT (GEM)	ER	5637/RT4/TCCSUP cell viability	TAM ↑growth inhibition	NA
Kashiwagi, 2016 [71]	CT (CIS)	AR	5637/647V/UMUC3 cell viability	AR-overexpression ↓sensitivity AR-knockdown/HF ↑sensitivity	NF-κB
Kameyama, 2017 [72]	CT (GEM)	AR	T24 cell viability	ENZ ↑sensitivity	cyclin D1
Ide, 2018 [73]	RT	AR	5637/647V/UMUC3 cell viability	AR overexpression/DHT ↓sensitivity AR-knockdown/HF ↑sensitivity	ATR, CHEK1, PARP1
Huang, 2019 [55]	CT (CIS, DOX, MMC)	AR	J82/TCCSUP cell viability J82 mouse xenograft	ASC-J9 ↑sensitivity	BAX, BCL2, p21
Long, 2019 [74]	CT (CIS)	ERβ	5637/T24 cell viability	Co-culture of CAF ↑ERβ expression ↓sensitivity	IGF1
Sekino, 2019 [75]	CT (CIS)	AR	RT112/UMUC3 cell viability	AR-overexpression ↓sensitivity	Uc.63+
Tyagi, 2019 [22]	CT (CIS)	AR	TCCSUP cell viability/migration/invasion	ENZ ↑sensitivity	EMT
Goto, 2020 [76]	CT (CIS)	ERβ	5637/647V/UMUC3 cell viability	ERβ-knockdown/TAM ↑sensitivity	β-catenin
Ide, 2020 [32] Ide, 2020 [77]	CT (CIS)	AR/ERβ	5637/647V/UMUC3 cell viability	AR/ERβ inactivate FOXO1 FOXO1-inactivation ↓sensitivity	FOXO1
Mizushima, 2020 [78]	BCG	AR	5637/MB49/UMUC3 cell viability	AR-overexpression/R1881 ↓sensitivity AR-knockdown ↑sensitivity	Rab27b
Jiang, 2021 [79]	CT (CIS)	AR	5637/UMUC3 cell viability	AR-overexpression/DHT ↓BXDC2 BXDC2-knockdown ↓sensitivity	BXDC2

↑: increase; ↓: decrease; BBN: N-butyl-N-(4-hydroxybutyl)nitrosamine; BCG: intravesical bacillus Calmette-Guérin immunotherapy; CAF: cancer-associated fibroblasts; CIS: cisplatin; CT: chemotherapy; DHT: dihydrotestosterone; EMT: epithelial-to-mesenchymal transition; ENZ: enzalutamide; DOX: doxorubicin; GEM: gemcitabine; HF: hydroxyflutamide; MMC: mitomycin C; MTX: methotrexate; NA, not available or not assessed; RT: radiotherapy; TAM: tamoxifen; TTP: thiotepa; VBL, vinblastine.

## Data Availability

Not applicable.
